# Transcriptomic insights into the biocontrol mechanism of *Trichoderma* spp. against Fusarium wilt in melon

**DOI:** 10.1007/s44154-026-00315-3

**Published:** 2026-06-22

**Authors:** Meriem Miyassa Aci, Polina C. Tsalgatidou, Konstantinos Krommydas, Anastasia Boutsika, Costas Delis, Ourania I. Pavli, Leonardo Schena, Antonios Zambounis

**Affiliations:** 1 Department of Agriculture, Università Degli Studi Mediterranea Di Reggio Calabria, Località Feo Di Vito, Reggio Calabria, 89124 Italy; 2 Department of Agriculture, University of the Peloponnese, Kalamata, 24100 Greece; 3 Hellenic Agricultural Organization - DIMITRA (ELGO - DIMITRA), Institute of Plant Breeding and Genetic Resources, Thessaloniki, 57001 Greece; 4 Laboratory of Plant Breeding, Department of Agriculture, Crop Production and Rural Environment, Fytokou St, University of Thessaly, Volos, 38446 Greece; 5 Hellenic Agricultural Organization - DIMITRA (ELGO - DIMITRA), Institute of Industrial & Forage Plants, Larissa, 41335 Greece

**Keywords:** Antimicrobial, Plant immunity, Transcriptomics, Weighted gene co-expression network analysis, Crop protection, Breeding for disease resistance

## Abstract

Fusarium wilt (FW), caused by *Fusarium oxysporum* f. sp. *melonis* (FOM), is a devastating disease severely impacting global melon (*Cucumis melo* L.) production. Biocontrol agents such as *Trichoderma* species, offer a sustainable alternative to chemical fungicides, yet their precise molecular mechanisms remain underexplored. In this study, we tested the antifungal efficacy of a commercial formulation combining two *Trichoderma* strains (*Trichoderma asperellum* ICC012 and *Trichoderma gamsii* ICC080) against FOM and investigated the molecular mechanisms underlying the melon roots *Trichoderma*-induced responses. Through phenotypic screening, RNA sequencing, and a Weighted Gene Co-expression Network Analysis (WGCNA) we demonstrated that *Trichoderma* pre-treatment significantly mitigates FW symptoms and orchestrates a robust defense response in melon roots. Our analysis revealed specific transcriptional reprogramming, including the upregulation of key hub genes such as NAC domain-containing protein 2, dehydration-responsive element-binding protein 1A (DREB1A), trihelix transcription factor GT-3b, and a caffeoylshikimate esterase-like encoding gene involved in lignin biosynthesis. Furthermore, critical pathways significantly enriched in *Trichoderma*-treated roots included phenylpropanoid biosynthesis, MAPK signaling pathway, and plant-pathogen interaction, alongside defense-related processes like zeatin and tryptophan biosynthesis and ABC transporter activity. These molecular reconfigurations highlight the complex signaling networks activated by *Trichoderma* spp., leading to enhanced immunity against FOM. Our findings provide crucial molecular insights into *Trichoderma*-mediated biocontrol, elucidating specific genetic and metabolic modulations in melon roots. This research paves the way for targeted breeding strategies and advanced industrial applications of *Trichoderma* spp. for effective and sustainable management of Fusarium wilt in melon crops.

**Supplementary Information:**

The online version contains supplementary material available at 10.1007/s44154-026-00315-3.

## Introduction

Melon (*Cucumis melo* L.) is an important vegetable species cultivated worldwide (FAO 2018; Yang et al. 2022), but its yield is critically threatened by various phytopathogens such as *Fusarium oxysporum* f. sp. *melonis* (FOM) (Leach & Currence) Snyder & Hansec, the causal agent of Fusarium wilt (FW) that causes severe losses reducing the crop productivity (Sestili et al. 2014). The management of this soil-borne pathogen, which can remain in the soil for many years through its chlamydospores, is very challenging and so far, the use of chemical fungicides has been the main and most effective control method (Gao et al. 2019; Jiang et al. 2019; Zhang et al. 2022). However, the biological control of soil-borne pathogens has already been recruited and greatly encouraged as alternative strategies for an ecologically friendly management of such diseases (Chen et al. 2021; Jiang et al. 2015, 2019; Zhang et al. 2022). In particular, application of microbial biological control agents (BCAs) was frequently employed to cope *F. oxysporum* diseases (Özkale et al. 2023; Zhang et al. 2022).

*Trichoderma* spp. are plant growth-promoting filamentous fungi with notable antimicrobial, antagonistic and mycoparasitic activities against a plethora of phytopathogens (Chen et al. 2017; Özkale et al. 2023; Zhang et al. 2022). As they act as BCAs enhancing the broad-range resistance in plants, they are widely used since registered as microbial fungicides (Zhang et al. 2022). Thus, various strains of *Trichoderma* species including* T*. *harzianum*, *T*. *atroviride* and *T. asperellum* have been effectively used to combat *F. oxysporum* (Corrales et al. 2010; Özkale et al. 2023; Yang et al. 2011), whereas *T*. *gamsii* 6085 was confirmed to have the ability to antagonize *F. graminearum* in rice (Matarese et al. 2012). In melon plants it was reported that *T*. *harzianum* can control FW by triggering the basal resistance and mitigating the hormonal imbalance induced by *F. oyxporum* (Martínez-Medina et al. 2010). Furthermore, these root-associated mutualistic BCAs can induce defense and immunity responses in plant hosts (Galletti et al. 2020; Moran-Diez et al. 2009; Pimentel et al. 2020; Romera et al. 2019; Salas-Marina et al. 2011).

RNA-sequencing (RNA-seq) technology is a widely adopted approach offering insightful information on plant–pathogen interactions allowing the in-depth transcriptome characterization during disease resistance or susceptibility responses of plants upon infection with pathogens (Yang et al. 2022; Nibedita and Jolly 2017). Using such advanced approaches, the differentially expressed genes (DEGs) involved in immunity responses can be elucidated along with the regulatory biological pathways that mediate these interactions (Tyagi et al. 2022). However, such host–pathogen interplays are typically driven by a complex transcriptional reprogramming engaging intricate and highly connected molecular networks to regulate defense responses (Delplace et al. 2022; Aci et al. 2024). In this line, the weighted gene co-expression network analyses (WGCNA) is a powerful tool that can be employed to uncover the main regulators (hub genes) that coordinate the transcriptional modulation of plant defense responses (Aci et al. 2024; Yu et al. 2017). In particular, hub genes within WGCNA represent highly connected nodes considered to play disproportionate roles in network stability and biological regulation (Amrine et al. 2015). In the context of plant defense, hub genes can act as central regulators of stress response networks, and modifications to their expression or sequence can have outsized effects on system stability and pathogen resistance outcomes (Amrine et al. 2015; Sari et al. 2019).

The transcriptional reprogramming involved in melon-FOM interactions was previously reported (Silvia Sebastiani et al. 2017; Martin 2011). The aim of the present study was to investigate the response of FOM-infected melon roots to *Trichoderma* treatment by investigating: i) the biocontrol efficacy of a commercial formulation containing *T. asperellum* ICC012 and *T. gamsii* ICC080, and ii) the defense responses mechanisms induced by *T. asperellum* and *T. gamsii* in FOM infected roots, by integrating RNA-seq approaches and WGCNA. Towards this, highly correlated regulatory networks were constructed and key hub DEGs were identified in response to *Trichoderma-*induced transcriptomic modulations in melon roots.

## Results

### Phenotypic screening and biocontrol effect of *Trichoderma* spp. on melon Fusarium wilt

**Fig. 1:**
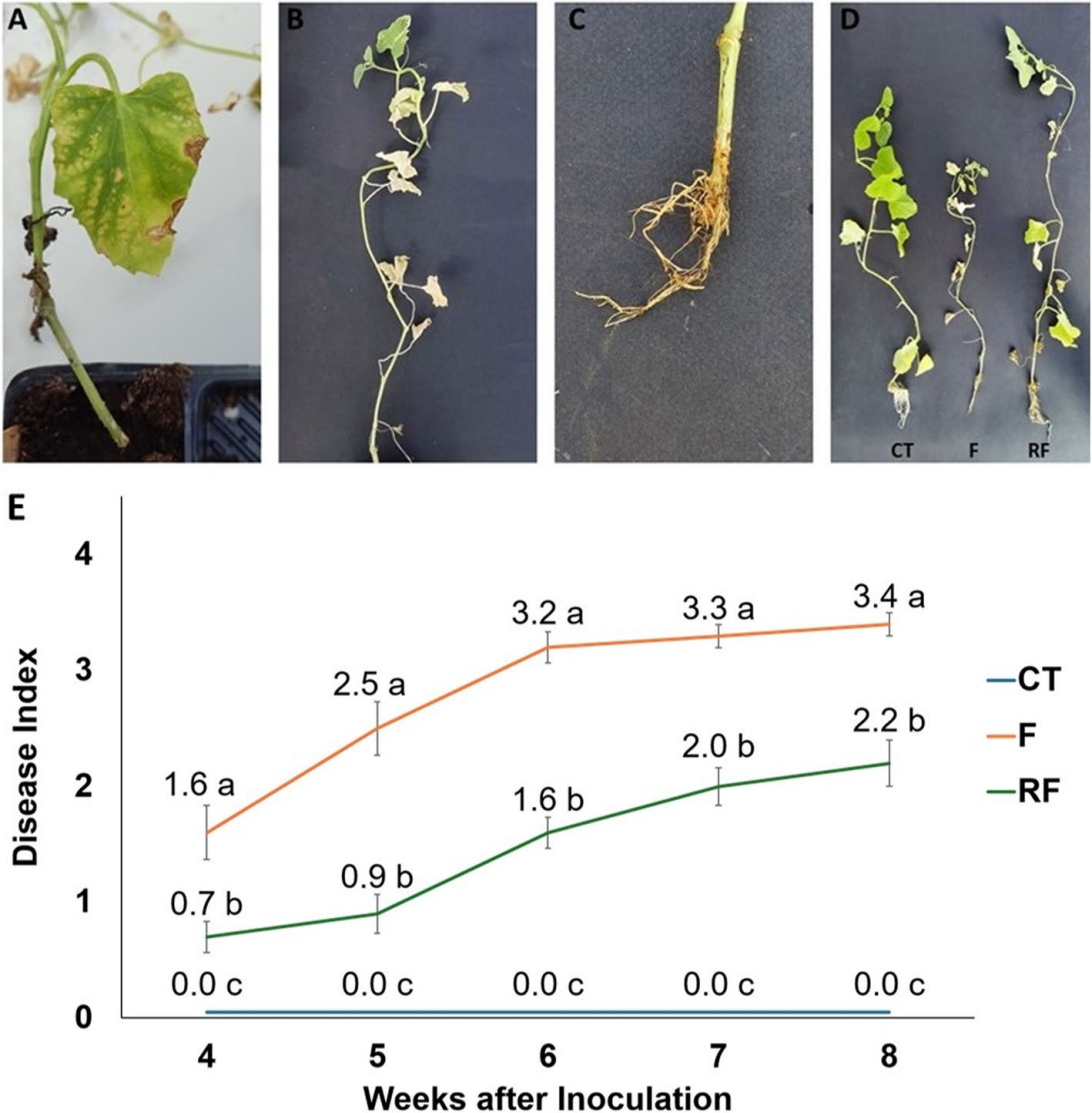
Representative symptoms and disease index of *Fusarium oxysporoum* f.sp. *melonis* (FOM) in *Cucumis melo* variety 'Xrisi-kefali' seedlings. Seedlings were artificially inoculated with the pathogen (F treatment) or treated with a *Trichoderma* formulation prior to pathogen inoculation (RF treatment). Untreated seedlings were used as the control (CT). **A**–**C** Symptoms observed in FOM-inoculated seedlings (F treatment): (A) leaf yellowing and necrosis on the leaf blade, (B) seedling wilting and defoliation, and (**C**) reduced root growth accompanied by reddish discoloration. **D** Comparison of seedlings inoculated with FOM (F treatment), pre-treated with Trichoderma and subsequently inoculated with FOM (RF treatment), and untreated control plants (CT treatment). **E** Disease index of F, RF, and CT seedlings recorded at four consecutive weekly intervals starting from the fourth week after FOM inoculation. Data represent mean ± SEM of biological replicates (*n* = 10 independent seedlings per treatment and time point). Different letters indicate statistically significant differences among treatments at the same time point according to one-way ANOVA followed by Duncan’s multiple range test (α = 0.05)

The biocontrol efficacy of *Trichoderma* strains (*T. asperellum* ICC012 and *T. gamsii ICC080)* against FOM on melon seedlings was assessed by monitoring the development of FW symptoms (Fig. 1). Typical FW symptoms, including leaf yellowing and necrosis with severe lesions on the leaf blade (Fig. 1A), seedling wilting and defoliation (Fig. 1B), as well as reduced root growth accompanied by reddish discoloration (Fig. 1C), were observed in FOM-inoculated seedlings (Fig. 1D; F treatment). The severity of these symptoms was markedly reduced in melon seedlings pre-treated with *T. asperellum* and *T. gamsii* prior to FOM inoculation (Fig. 1D; RF treatment). Weekly assessment of the disease index starting from the fourth week after FOM inoculation, revealed a significantly lower disease severity in *Trichoderma*-treated seedlings over a four-week period (Fig. 1E). As expected, non-inoculated control plants (CT treatment) did not exhibit any disease symptoms.

The protective as well as the plant growth promoting effect of *Trichoderma* formulation was further evident by monitoring various growth-related parameters at eight weeks after the pathogen inoculation. Beyond disease suppression, seedlings treated with the *Trichoderma* formulation (RF treatment) exhibited a significantly greater shoot height, stem diameter, and number of internodes compared with seedlings of F treatment (Table S1). In contrast, an opposite trend was observed for defoliation percentage. In the F treatment a significant portion of the leaves (~79%) was lost, while defoliation in RF seedlings was limited to approximately 28%. Finally, disease incidence, expressed as the percentage of symptomatic plants, reached 100% in the F treatment, while RF seedlings showed a 40% percentage of plants with typical wilting symptoms (Table S1).

### Analysis of transcriptome and DEGs identification

**Fig. 2:**
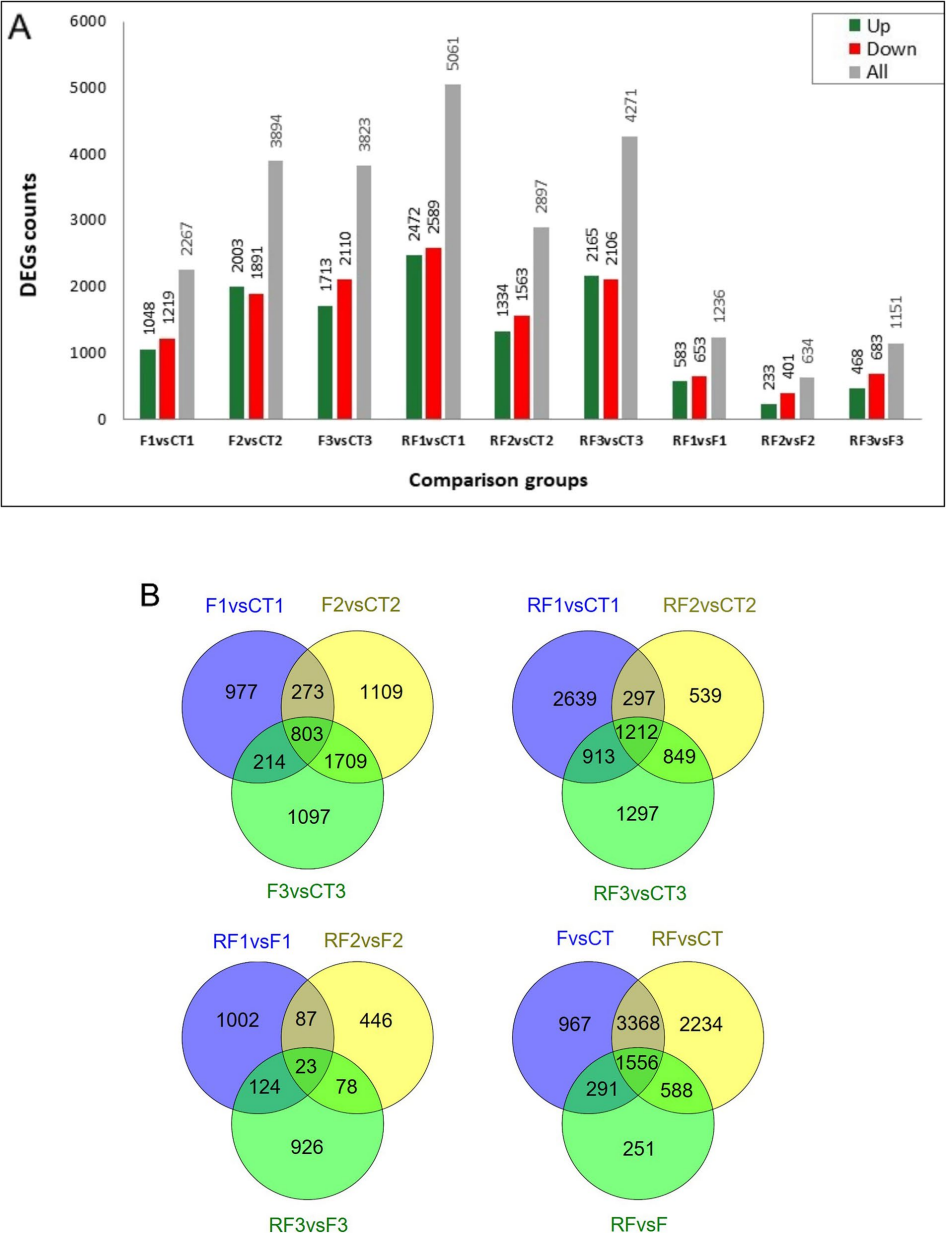
Summary statistics of the DEGs identified across the nine comparison groups. **A** Number of up- and down-regulated DEGs for each comparison group at the three time points, **B** Venn diagrams performed to filter for the unigenes. The number of DEGs identified at each time-point within each group were compared and a total of 6182, 7746 and 2686 unigenes were identified in FvsCT, RFvsCT and RFvsF comparisons, respectively. Then, the unigenes of all groups were compared in a final Venn diagram where a total of unique 9254 genes were identified for the downstream analyses

To explore the regulatory mechanisms of the specific *Trichoderma* strains in the biocontrol of Fusarium wilt in melon, an RNA-seq analysis was performed on i) *Trichoderma* spp. pre-treated melon root samples following by FOM inoculation (RF treatment) and ii) on samples inoculated only with the pathogen (F treatment). Gene expression profiles were determined against non-inoculated control samples (CT) across three time points (1, 2, and 3 days after inoculation). A total of approximately 944 million clean reads (on average ~34 million reads per sample) were generated in the transcriptome sequencing and the efficacy of their mapping to the melon reference genomic assembly reached more than 88% (Table S2). DEGs |log2foldchange|≥ 1, with an FDR-corrected *p*-value ≤ 0.05 were identified across nine time-specific comparison groups (Fig. 2A). The correlation between the up and downregulated DEGs is shown also to the relevant volcano plots (Fig. S1). Among those DEGs, the highest number of upregulations was revealed in RF1vsCT1 (2472) and to a lesser extend in RF3vsCT3 group (2165). Notably, at 1 and 3 days after pathogen inoculation, a higher number of DEGs was induced in melon roots treated with *Trichoderma* spp. and FOM as compared with roots inoculated only with the pathogen. This suggests that these specific *Trichoderma* strains can significantly induce with a time-dependent profile the differential expression of some genes that may potentially be related to melon FW defense. Furthermore, Venn diagrams analysis for the identified DEGs in each treatment comparison among all time points, 6182, 7746 and 2686 unigenes were retrieved from FvsCT, RFvsCT and RFvsF comparisons at all time-points, respectively (Fig. 2B). A non-redundant set of 9254 differentially expressed genes was thus identified (Fig. 2B, Table S3). This selection was critical for subsequent WGCNA, serving to mitigate biological noise and focus on core transcriptional responses to the experimental conditions. By utilizing this refined gene set, we aimed to enhance the robustness and biological interpretability of the constructed co-expression modules, thereby accurately delineating regulatory networks underlying *Trichoderma*-mediated biocontrol mechanisms in melon roots.

To validate the RNA-seq data, a linear regression model was employed to track the correlation between transcriptomic data and RT-qPCR log2foldchange values for the F2vsCT2 and RF2vsCT2 comparison groups using six randomly selected DEGs. The expression patterns of the selected genes were comparable to the RNA-seq results showing a linear fit line with a high coefficient of determination (Fig. S2).

### Identification of co-expressed modules using the WGCNA approach

**Fig. 3:**
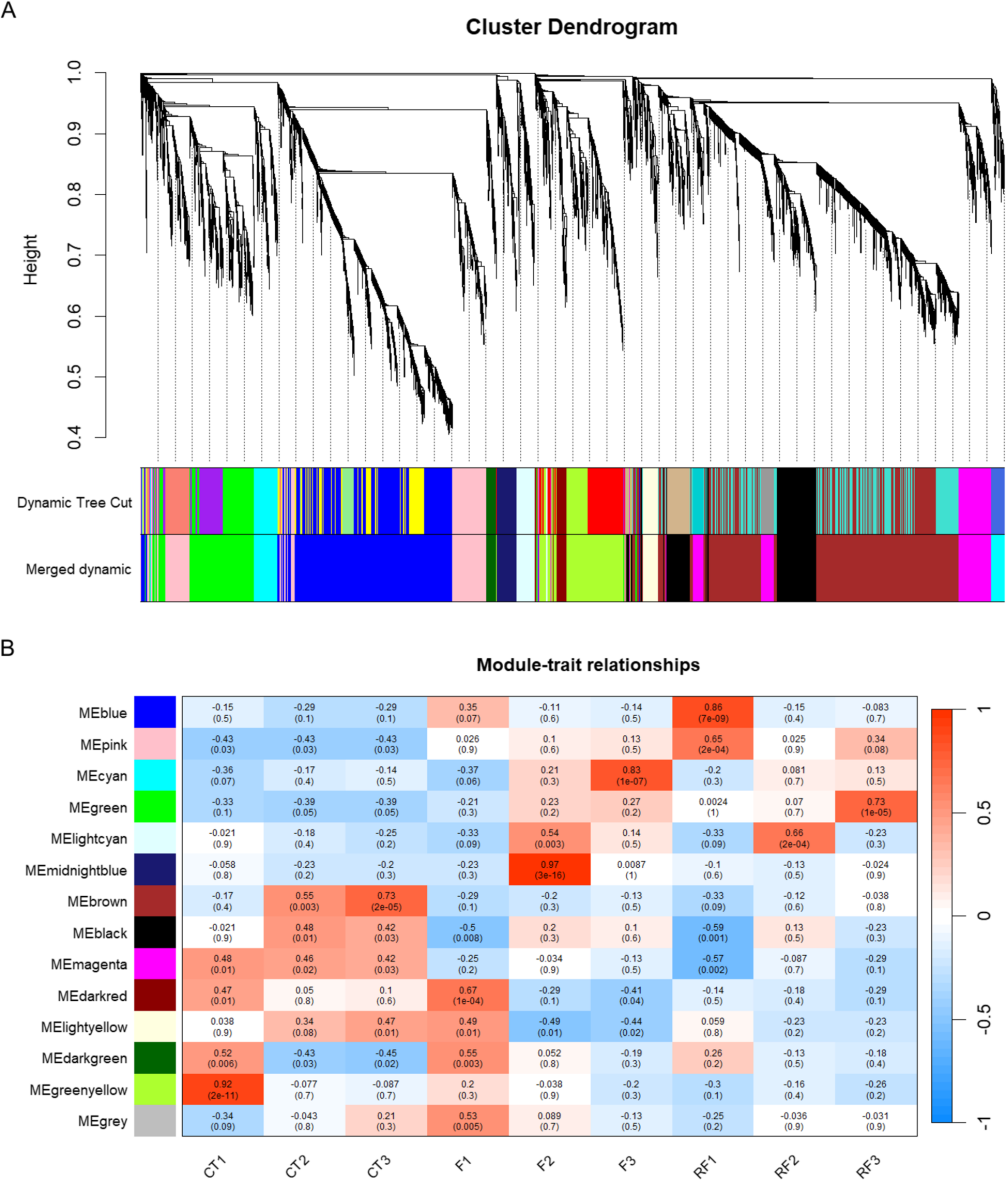
WGCNA analysis and module trait relationships. **A** The cluster dendrogram of 9254 unigenes; each branch of the dendrogram represented one gene, and every color below represents one co-expression module. Gray module grouped the genes that were not part of any module. A total of 14 merged co-expression modules were obtained. **B** Correlation between modules and different treatment at each time point. In the heatmap of correlations, negative number (blue) indicates negative correlation between eigengenes and modules, and positive number (red) indicates positive correlation, numbers between brackets represent the significance

The WGCNA was conducted to uncover key modules and hub genes using 9254 unique DEGs. Based on the soft power = 20 (Fig. S3A), the Dynamic Tree Cut with 100 of minModuleSize grouped the candidate genes into 24 co-expression modules, which were ulteriorly merged into fourteen modules filtering for at least 80% similarity (Table 1, Fig. S3B, Fig. 3A). The grey module refers to genes that are not divided into specific modules and will be eliminated in subsequent analysis.

**Table 1:** DEGs number within each module resulting from the weighted gene co-expression network analysis

Module	DEGs Number	Module	DEGs Number	Module	DEGs Number
Black	757	Darkgreen	114	grey	6
Blue	1850	Darkred	116	lightcyan	215
Brown	2316	Green	859	lightyellow	162
Cyan	423	greenyellow	782	magenta	691
Midnightblue	220	Pink	743

### Correlations analysis between treatments and modules

To determine the most interesting modules involved in melon responses to FOM inoculation alone (F treatment) or in combination with *Trichoderma* pre-treatment (RF treatment), the correlation between 14 modules and differentially expressed genes was analyzed (Fig. 3B). Each group of data in the Fig. 3 represents the correlation coefficient (ME) and significance *p*-value between the module and sample-treatment (within parentheses). The larger the absolute value, the greater the correlation, with blue indicating negative correlation and red indicating positive correlation. Significant modules were filtered for a ME ≥ 0.70. The midnightblue (ME = 0.97, *p* < 0.0001) and the cyan (ME = 0.87, *p* < 0.0001) modules, with 220 and 423 genes, respectively, were highly correlated to FOM infection at the second and third day after infection (F2 and F3 treatments), respectively. Whereas the blue (ME = 0.86, *p* < 0.0001) and green (ME = 0.73, *p* < 0.0001) modules, enclosing 1850 and 859 genes, respectively, were highly correlated to RF treatment at the first and last day (RF1 and RF3 treatments), respectively (Fig. 3B).

### GO and KEGG pathways enrichment analysis of the key modules

**Fig. 4:**
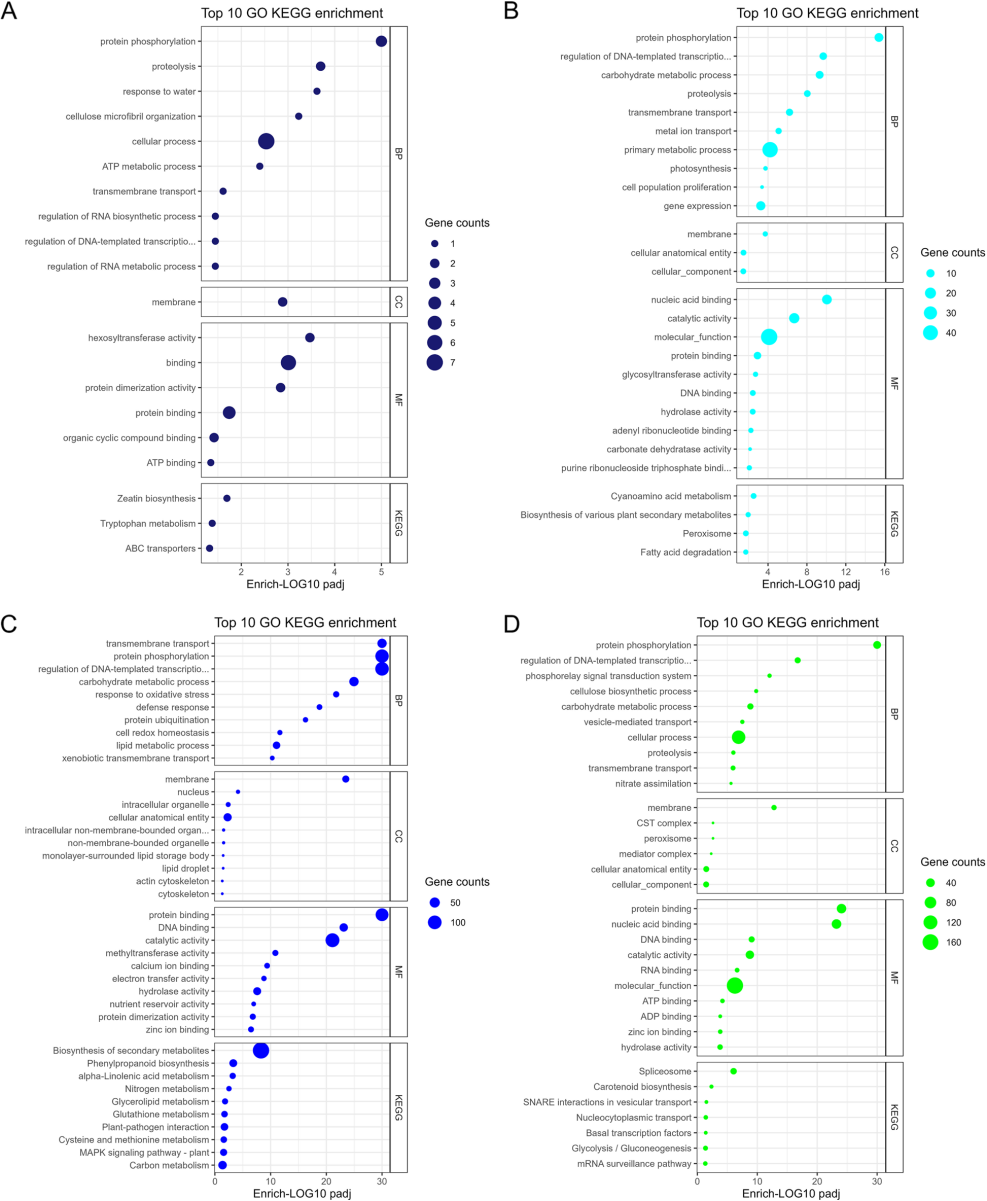
Top 10 enriched GO terms and KEGG pathways for the differentially expressed genes (DEGs). **A**) in the midnightblue module; **B**) in the cyan module; **C**) in the blue module, and **D**) in the green module

To further explore the function of the module of interest, the genes within each module were mapped to the GO and KEGG pathways databases and only the top 10 GO terms and KEGG pathways for each module were plotted (Fig. 4A, B, C, D). In the midnightblue and cyan modules, highly correlated with the pathogen at the second and third day of infection (F2 and F3, respectively), many genes were significantly enriched in protein phosphorylation, proteolysis, transmembrane transport and the regulation of both RNA biosynthesis and DNA-template transcription biological processes, and in binding molecular functions. The KEGG pathways significantly enriched in the midnightblue module regarded zeatin and tryptophan biosynthesis and ABC transporter (Fig. 4A), and in cyanoamino acid metabolism, peroxisome and fatty acids degradation in the cyan module (Fig. 4B). Among the GO terms significantly enriched in the blue and green modules, strongly correlated to the RF combination treatment at the first and third day after inoculation (RF1 and RF3, respectively), we found the defense response and response to oxidative stress (blue module) (Fig. 4C), as well as cellulose biosynthetic process and nitrate assimilation biological processes (green module) (Fig. 4D). Furthermore, many genes in both modules were significantly enriched in DNA, RNA and protein binding, catalytic activity as well as calcium and zinc ions binding molecular functions (Fig. 4C, D). With respect to the other modules, the blue and green ones revealed the involvement of many cellular components such as membrane, nucleus, cytoskeleton, intracellular organelle, and peroxisome (Fig. 4C). As well for the KEGG pathway enrichment analysis, many plant resistance-related pathways were significantly enriched in the blue module, such as the phenylpropanoid biosynthesis, alpha-linoleic acid metabolism, plant – pathogen interaction and MAPK signaling pathways (Fig. 4C), and in the green one such as carotenoid biosynthesis pathway (Fig. 4D).

### Key modules regulatory networks analysis and hub genes identification

Filtering for gene trait significance (GTS) and module membership (MM) > 0.75 we selected the top fifty genes with the highest |log2FC| values for each treatment with respect to its control in the four critical modules (Fig. S4A, S5A, S6A, S7A). The description and the expression patterns of the selected genes across the nine comparisons are presented in Fig. S8A-D.

The fifty identified genes from each module were then used as bait genes in the construction of the regulatory networks to evaluate their connection degree within each module-network and identify among them the hub genes. For each module, we identified the top 10 most connected genes in the regulatory network and reported the genes’ parameters. For the midnightblue module (Fig. S4B, Table S5) the regulatory network was composed of 105 nodes connected by 557 edges. The top 10 connected nodes were ribosomal RNA encoding genes (28S ribosomal RNA types), suggesting a key role of this module in the melon roots transcriptional reprogramming after FOM infection.

The cyan regulatory network (Fig. S5B) was composed of 172 nodes connected by 733 edges. Among the top 10 most connected genes we found the LOC103492895 (glutathione S-transferase F13, GST-F13), LOC107991526 (pyridoxal kinase-like, PDXK) and LOC103484706 (elongation of fatty acids protein 3-like, ELO3L) with 58, 42 and 37 connections, respectively. The remaining seven hub genes were uncharacterized loci (Table S6).

In the blue module network (Fig. S6B) a total of 421 nodes connected by 2275 edges were observed. Among the top 10 most connected genes we identified four transcription factors (TFs): LOC103489142 (NAC domain-containing protein 2, NAC2), LOC103486288 (trihelix transcription factor GT-3b), LOC103484041 (dehydration-responsive element-binding protein 1 A, DREB1A), and LOC103496341 (heat stress transcription factor A-4c-like, HSF A4cL). Along with LOC103489142 (NAC2) and LOC103484041 (DREB1A), the loci LOC103485032 (caffeoylshikimate esterase-like, CSE), and LOC103483929 (late embryogenesis abundant protein D-29, LEA-D29) showed the highest connectivity levels within the blue regulatory network (210, 202, 230, and 198 connections, respectively) (Fig. S6B, Table S7).

The analysis of the green module regulatory network showed the presence of 303 nodes connected by 1635 edges. Among the hub genes identified in this module we found LOC103486099 (splicing factor U2AF-associated protein 2), LOC103484043 (two-component response regulator ARR14-like), LOC103482610 (kunitz trypsin inhibitor 5, KTI 5), LOC103482604 (miraculin-like, MLP), LOC103489800 (transcription initiation factor IIF subunit alpha, TFIIF-a), LOC103488440 (eukaryotic translation initiation factor 3 subunit A-like, eIF3), and LOC103486512 (transcription factor MYB27-like) (Fig. S7B, Table S8).

**Fig. 5:**
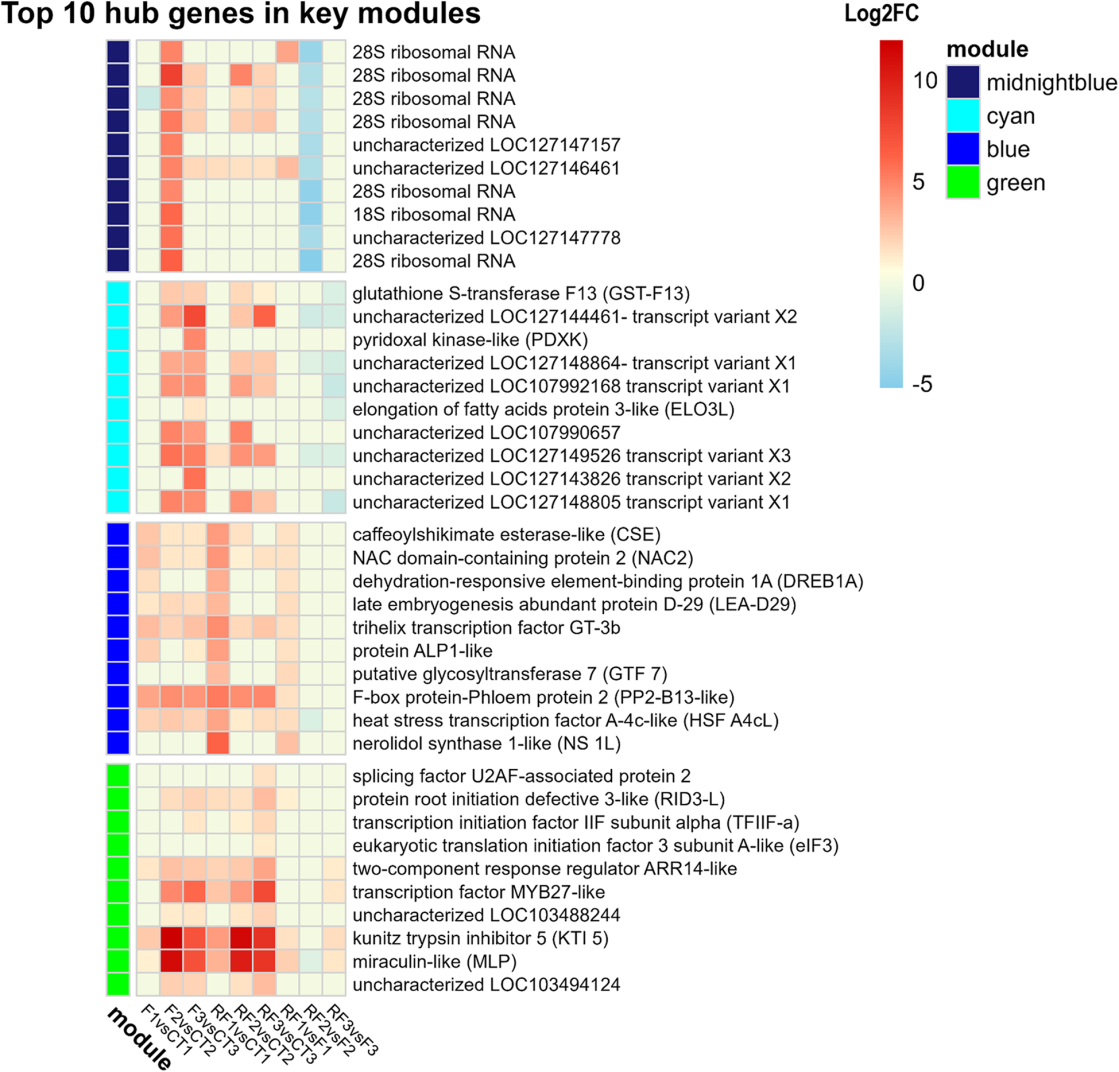
Heatmap of the differential expression (log_2_ (FC) >|1|, FDR < 0.05) of the 10 hub genes identified in the four key modules. Top 10 hub genes are represented by their respective colors midnightblue, cyan, blue and green, across the nine comparison groups

The expression patterns of the top 10 hub genes in each module across all treatment comparisons were presented in a heatmap (Fig. 5, Table S9). Among the most evident results in the midnightblue module, the hub genes were upregulated in response to FOM infection at the second day in respect to the control treatment (F2vsCT2). The expression trend was inverted in presence of *Trichoderma* treatment (RF2vsF2) compared to the FOM inoculation alone. The expression patterns for the hub genes identified in the other key modules were almost the same. These results suggest that *Trichoderma* formulation could target the transcriptional machinery regulating in almost cases the genes activated by the presence of FOM inoculation alone.

### FOM expressed genes

We also analyzed the pathogen transcripts obtained within our data sets. Across all three time points (D1 to D3), the comparison between F and RF treatments (Fig. S9) shows a consistent and pronounced reduction in gene expression under RF treatment. Many of the top expressed FOM genes display strong repression when *Trichoderma* formulation is present, often approaching near-zero expression levels (Table S10). Functionally, the inhibited genes include several ribosomal proteins (e.g. 50S and 60S subunits), suggesting a reduction in protein synthesis capacity, as well as key metabolic and mitochondrial enzymes such as ATP synthase (*FOMG_00985*), malate dehydrogenase (*FOMG_01936*), cytochrome c peroxidase (*FOMG_00795*), and aldehyde dehydrogenase (*FOMG_01701*), indicating a potential suppression of respiratory and energy metabolism pathways. Importantly, several genes associated with pathogenicity and plant cell wall degradation, including xyloglucanases (*FOMG_09724*), pectate lyases (*FOMG_09832*), endoglucanases (*FOMG_03083*, *FOMG_03485*, *FOMG_07426*, *FOMG_14546*, *FOMG_15155*) and exoglucanases (*FOMG_01181*), are also markedly reduced in expression, particularly at later time points. Together, these patterns suggest that *Trichoderma* exerts a broad inhibitory effect on FOM, impacting core cellular processes (translation and respiration) as well as virulence-associated functions. While formal differential expression analysis is required to statistically confirm these effects, the expression profiles strongly indicate that *Trichoderma* includes a sustained transcriptional suppression of key growth and pathogenicity-related genes in FOM.

## Discussion

Melon is a significant fruit crop that is severely threatened by FOM leading to reduced crop production (Sestili et al. 2011). Although extensive melon breeding efforts aimed at increasing FW tolerance have been undertaken so far, the underlying resistance mechanisms remain unclear (Yu et al. 2017). In addition, fungal BCAs are becoming an effective alternative management strategy in crop protection field worldwide (Kumari et al. 2024). Among these, fungal species belonging to the genus *Trichoderma* are employed as potential BCAs against a wide spectrum of soil-borne pathogens (Guzmán-Guzmán et al. 2023; Pimentel et al. 2020; Ζhang et al. 2020b; Zhang et al. 2022). For example, *T. asperellum* M45a was effective against FW in watermelon, whereas *T. gamsii* 6085 showed an antagonistic effect against *F. graminearum* in rice (Matarese et al. 2012; Ζhang et al. 2020b). In addition to promoting plant nutrient uptake and growth and exerting mycoparasitic and antagonistic biocontrol activity, various *Trichoderma* species may induce plant defense mechanisms upon pathogen challenge (Chen et al. 2021; Zhang et al. 2022; Zhao et al. 2021; Mayo et al. 2016). For instance, *T. asperellum* TS-12 and TS-39 considerably enhanced the disease resistance of tomato plants against *F. oxysporum* f*.*sp.* lycopersici* (FOL) by intensively triggering the expression levels of defense-related genes (Komy et al. 2016). In addition, a differential expression of many genes that could be associated with transduction and systemic resistance against *F. oxysporum* f.sp. *niveum* (FON) was observed at transcriptomic level in *T*. *asperellum* M45a-treated watermelon roots (Zhang et al. 2022). However, the accurate regulatory mechanisms and the hub genes involved beyond the elicitation of *Trichoderma-*induced plant defense responses, particularly upon pathogen infection, still need to be further investigated.

In this study, the efficiency of a commercially available formulation that contains selected strains of *Trichoderma* spp. (*T. asperellum* ICC012 and *T. gamsii* ICC080) was initially evaluated for its biocontrol effect on FOM infected melon roots. The results revealed that the pre-treatment with this fungal BCA significantly mitigated the FW symptoms upon subsequent FOM inoculation. Monitoring of various growth-related parameters, along with disease index and disease incidence, clearly highlighted the evident protective role of these *Trichoderma* species against FW in melon. Transcriptomic analysis revealed that specific DEGs and associated pathways may be associated with the disease resistance mechanisms and immune responses to FOM infection. Beyond disease suppression, the increased shoot growth observed in RF seedlings is consistent with the widely reported plant growth-promoting activity of *Trichoderma* spp. in the rhizosphere, which can enhance nutrient availability (e.g., mineral solubilization/Fe-chelation) and stimulate plant development through multiple direct and indirect mechanisms (Chen et al. 2025; Contreras-Cornejo et al. 2024). Such effects are often linked to the production of bioactive metabolites and VOCs and to modulation of plant hormonal signaling (e.g., auxin-related pathways), ultimately improving root architecture and overall plant vigor, although responses can be strain- and context-dependent (Chen et al. 2025; Contreras-Cornejo et al. 2024).

Our transcriptomic analysis revealed a strong suppression of FOM gene expression profile in the presence of the *Trichoderma* BCA across all three time points, indicating its direct biocontrol capacity. Notably, the repressed genes included multiple ribosomal proteins, key mitochondrial and metabolic enzymes, and carbohydrate-active enzymes associated with pathogenicity and plant cell wall degradation, suggesting impaired translational capacity, disruption of respiratory metabolism and cellular energy production, as well as suppression of FOM pathogenicity. Such suppression of primary metabolic processes indicates that *Trichoderma* may compromise fungal viability at a fundamental physiological level. These findings are consistent with the results reported by Cheng and colleagues where the biocontrol agent *Trichoderma hamatum* induced widespread transcriptional reprogramming in the *F. graminearum* pathogen, including downregulation of genes involved in ribosome biogenesis, energy metabolism, mitochondrial function, protein synthesis and virulence (Cheng et al. 2026). Similarly, significant transcriptomic changes were also reported for the *Fusarium virguliforme* when challenged with *Trichoderma afroharzianum* Th19A and Th4 BCAs (Pimentel et al. 2025). A significant number of genes related to metabolic and carbohydrate metabolic processes, virulence and resistance to antifungal agents, indicating the strong biological control capacity of the *Trichoderma* species (Pimentel et al. 2025).

Considering the dynamic nature of the fungal infection process, WGCNA was initially applied and co-expressed gene clusters (modules) significantly associated with pathogen resistance in various crops were successfully identified (Aci et al. 2024; Cui et al. 2022; Kumar et al. 2022). In the present study, WGCNA was performed to gain insights into the specific gene expression patterns that actively regulate the *Trichoderma* spp.- induced responses during FOM-melon interactions. The analysis led to the identification of four significant time-dependent co-expression modules, all positively correlated with either the F treatment at 2 and 3 days (midnightblue and cyan modules, respectively), or with the RF treatment at 1 and 3 days (blue and green modules, respectively).

It is worth noting that the hub genes identified in the midnightblue key module were, ribosomal RNA- encoding genes, mainly of 28S ribosomal RNA types that were all up-regulated in response to F treatment. This suggests that after two days with FOM, a dynamic transcriptional reprogramming of ribosomal activity is triggered in melon root cells, which may regulate any melon defense responses or mediate pathogen-induced susceptibility. Functional analysis revealed that the DEGs within midnightblue module were mainly involved in both cellular and binding processes, which further supports the hypothesis that this module is involved in the pleiotropic and complex transcriptional regulation of melon roots in response to FOM. Interestingly, the expression patterns of the genes involved in this module were inverted when FOM was inoculated after *Trichoderma* pre-treatment. These results highlight the interference of *Trichoderma* spp. on the signal interplay between FOM and melon roots, consequently limiting transcriptome reprogramming. This interference may result of i) *Trichoderma* spp. competition/antagonism against FOM, ii) *Trichoderma* spp. plant-induced systemic resistance, iii) *Trichoderma* spp. plant hormone modulation, or iv) a combination of these effects (Ali et al. 2024; Dodds et al. 2024).

Notably, in the cyan module, which was significantly correlated to FOM infection after three days (treatment F3), many non-coding transcripts annotated in the genome as long non-coding RNAs (lncRNAs) were upregulated and were among the hub genes with the highest |Log2FC| (FDR ≤ 0.05). Recent studies have shown that these RNAs with a length of more than 200 nucleotides are key regulators of plant immunity in response to pathogens (Bhar 2023; Huang et al. 2023; Samarfard et al. 2022; Zhang et al., 2020a), and have been previously identified as DEGs at different immunity stages in response to pathogens’ infection (Joshi et al. 2016; Rosli et al. 2021; Xin et al. 2011; Zhu et al. 2014). Among their various pleiotropic and underestimated roles in shaping the overall plant immune responses, lncRNAs have been implicated in regulating ROS accumulation through modulation of nearby genes in the host genome (Cui et al. 2019), in calcium influx triggering downstream signaling upon pathogen infection (Yang et al. 2021), and in altering the defense-related gene expression mainly by targeting or regulating transcription factors (TFs) (Seo et al. 2017; Liu et al. 2022b). Notably, two cotton lncRNAs were reported to be induced after infection with *Verticillium dahliae* or *Botrytis cinerea*, by repressing the expression of two lipoxygenase-encoding genes involved in the JA pathway, thereby attenuating plant resistance (Zhang et al. 2018). In our study, we speculate that the constitutive upregulation of lncRNAs within the cyan module, that is related with the later responses of melon roots upon FOM inoculation, may play a remarkable role in modulating the expression of other genes during transcriptional preprograming. Furthermore, two uncharacterized DEGs (LOC127144461 and LOC127150244), showing homology to long terminal repeat (LTR) retrotransposon gag proteins, were highly upregulated within this module. Transposable elements (TEs) have been proposed to mediate to the diversification of fast-evolving disease resistance genes. In particular, retrotransposons, which are the most abundant group of TEs, might be transcriptionally activated upon plant challenge with biotic elicitors leading to stress-mediated responses and facilitating host adaptation (Galindo-González et al. 2017; Zervudacki et al. 2018). Among the protein-coding DEGs inside the cyan module, a gene encoding pyrixidal kinase (PDXK) displayed high overexpression and a distinct expression profile compared to the other comparison groups. This gene is involved in the biosynthesis of vitamin B6 via the plant salvage pathway, which is well recognized as an essential antioxidant in stress responses (Samsatly et al. 2020). Previously, a bacterial effector from *Xanthomonas oryzae* pv. *oryzicola* has been reported to inhibit vitamin B6 biosynthesis to exchange infection in rice (Liu et al. 2022a).

The blue module was positively correlated with the *Trichoderma*-induced early defense responses in melon roots against FOM inoculation. Various TF-encoding DEGs with high connectivity within the regulatory network were among the top 10 highly upregulated hub genes. In line with this, the biological process “regulation of DNA-templated transcription” was highly enriched into the blue module. Specifically, DEGs encoding members of the NAC, DREB1A, *Trihelix* GT*−3b,* and HSF families were highly upregulated in the RF1 treatment, suggesting that these TFs are actively involved in the *Trichoderma*-induced transcriptional programming towards the activation of melon defense responses. It is known that these TF families play a crucial role in the orchestration of defense mechanisms in plants upon pathogen challenge (Aci et al. 2024; Noman et al. 2019; Tsalgatidou et al. 2024; Yang et al. 2022; Zhang et al. 2021a, b; Tsuda et al. 2015). For example, a member of trihelix GT-3b TF family has been associated with the regulation of defense response against *F. graminearum* in maize (Zhang et al. 2021a, b), whereas a pathogen-induced putative NAC TF has been shown to modulate leaf rust resistance in barley (Chen et al. 2021). Furthermore, upregulation of a DREB1A enhances tolerance to biotic stress in transgenic potato (Charfeddine et al. 2015).

Plant inducible immunity includes the accumulation of a set of defense-related proteins following a pathogen attack. Among them, lignin and phytoalexin associated pathways play a central role in modulating host defense against fungal pathogens. Into the blue module, KEGG pathways such as “biosynthesis of secondary metabolites”, “phenylpropanoid biosynthesis”, “plant pathogen interaction”, and “MAPK signaling pathway” were significantly enriched. Particularly, among the top 10 highly upregulated hub genes, a gene encoding caffeoyl shikimate (CSE) was identified. CSE contributes in the regulation of lignin biosynthesis upon fungal attack and has been previously reported to be involved in the immune defense of cucumber against *Podosphaera xanthii* (Yu et al. 2022). It is known that lignin production is a branch of the phenylpropanoid pathway, a main metabolic hub responsible for the biosynthesis of secondary metabolites and phytoalexins that plants employ to counter fungal challenges (Ninkuu et al. 2022).

Moreover, a DEG encoding a lysin motif receptor kinase (LYK5) was also upregulated inside the blue module. This receptor gene plays a major role in chitin recognition and, along with a *CERK1* gene mediates chitin-induced plant immune signaling in *Arabidopsis* (Cao et al. 2014). In addition, a gene encoding phloem protein 2 (PP2), related to phloem-based defense in plants through binding to various carbohydrates and is induced upon pathogen attack, was upregulated into the blue module (Bobbili et al. 2023). This gene contains a highly conserved lectin domain along with an F-box motif in its N-terminal domain, suggesting a multifunctional role in defense signaling. Among the top 10 hub genes in this module, a nerolidol synthase 1-like (NS-1L) encoding gene was also found. NS-1L has been previously reported to act as a volatile signal that enhances defense responses in tea plants against pathogens (Chen et al. 2020). Furthermore, a DEG encoding subtilisin-like protease SBT3.4 (subtilase) was among the 50 hub genes with the highest |Log2FC| values (FDR ≤ 0.05) in the blue module. In the last years, subtilases have been gaining high attention regarding their involvement in plant immunity responses against pathogens (Figueiredo et al. 2018).

The DEGs belonging to the green module were highly correlated to the transcriptional modulation of the *Trichoderma*-induced defense responses that offer a biocontrol effect against FOM in melon roots to the later stage upon infection. Notably, a DEG encoding a two-component response regulator ARR14-like was among the top 10 hub genes which were upregulated inside this regulatory module. ARR14-like gene has emerged as a new SA/JA crosstalk regulator involved in cytokinin-mediated responses against the necrotrophic fungal pathogen *B. cinerea* in *Arabidopsis* (Falconieri et al. 2022). Furthermore, a gene encoding KTI 5 was also detected in this module; previously, this plant proteinase inhibitor family showed both antifungal activity and elicited defense responses in tobacco (Huang et al. 2010). Among the other hub genes inside the green module, a MLP- encoding gene (major latex protein) was found. This gene belongs also to Kunitz superfamily possessing trypsin inhibitory activity and is involved in plant defense (Huang et al. 2010). Similarly, an antifungal miraculin-like protein (Selvakumar et al. 2011), called sativin, depicted antifungal activity against *F. oxysporum* in legumes (Ye et al. 2000). MYB TFs play also a pivotal role in disease resistance against pathogens (Yu et al. 2023; Biswas et al. 2023). In this study, a MYB27 encoding gene was detected among the top 10 hub genes into the green module. Additionally, the induction of two hub genes encoding TFIIF-a and elF3 factors suggest their crucial roles in the regulation of *Trichoderma*-induced responses in melon roots after FOM infection. It is interesting to note that an aquaporin (AQP)-encoding DEG was also highly correlated in the green module. These genes encode membrane channel proteins that influence defensive signaling pathways during plant-pathogen interactions (Wang et al. 2018).

Examining the module-trait correlations in the context of disease severity, we further elucidate the causal relationship between co-expression modules and phenotypic outcomes. The midnightblue module, significantly correlated with the F treatment, reflects a transcriptomic state associated with active pathogen challenge, whereas the blue module is exclusively linked to the early *Trichoderma*-induced response. Strikingly, the blue module is significantly enriched for defense-related KEGG pathways, such as phenylpropanoid biosynthesis and MAPK signaling, suggesting that the specific activation of these co-expression networks underpins the mitigation of wilting symptoms. The identification of hub genes within the blue module, including CSE and NAC2, which regulate lignin biosynthesis and stress tolerance respectively, provides a mechanistic explanation for the reduced disease severity observed in Trichoderma-treated plants. This integration of module traits with phenotypic data reinforces the interpretation that these specific transcriptional reprogramming events are drivers of the resistance phenotype.

A deeper analysis of the dynamic nature of the *Trichoderma*-FOM-melon interaction reveals that the observed transcriptomic changes comprise both downstream responses to disease suppression and active regulatory shifts driving induced resistance. For instance, the upregulation of 28S ribosomal RNA and the activation of lncRNAs in the midnightblue and cyan modules (F treatment) appear to represent a dynamic, yet potentially pathogen-induced transcriptional reprogramming. The inversion of these expression trends under *Trichoderma* pre-treatment suggests that the transcriptome changes associated with FOM infection are downstream consequences that are effectively mitigated by the biocontrol agent. Conversely, the blue module, positively correlated with the RF treatment, delineates the active regulatory machinery primed by *Trichoderma*. The significant enrichment of plant-pathogen interaction and MAPK signaling pathway in this module indicates a proactive defense readiness. Furthermore, the marked upregulation of specific TFs (NAC2, DREB1A, Trihelix GT-3b) and biosynthetic genes (CSE, NS-1L) underscores a host-driven genetic reprogramming that establishes resistance prior to substantial pathogen ingress, rather than merely reacting to it.

While this study demonstrates promising control efficacy against FW in melon, it is important to acknowledge that the long-term ecological consequences of microbial biocontrol agents’ application on soil microbial community structure and dynamics have already been proven (Aci et al. 2025). More specific evidence indicates that *Trichoderma* inoculation can significantly alter resident soil fungal communities, including reduced fungal alpha-diversity and shifts in relative abundances over time, depending on soil type and persistence of the inoculant (Clagnan et al. 2023; Leal et al. 2023). Consequently, the introduction of biocontrol agents may drive restructuring of the rhizosphere microbiome or increase beneficial species abundance, potentially creating suppressive states that sustain pathogen control or conversely alter competitive equilibria in complex ways (Bandara and Kang 2024; Zhang et al. 2021a, b). Recognizing this knowledge gap is critical, as understanding how *Trichoderma*-mediated shifts persist and interact with indigenous *Fusarium* populations could inform optimization strategies for durable disease management while safeguarding soil biodiversity.

It should be noted that our experimental design focused on *Trichoderma* pre-treatment followed by pathogen inoculation, which reflects typical field application practices where BCAs are applied preventively to establish in the rhizosphere before pathogen challenge (Harman et al. 2004; Shoresh et al. 2010). The biocontrol efficacy of *Trichoderma* largely relies on priming plant defense responses and establishing colonization in the root system prior to pathogen attack, as effective endophytic colonization is essential for inducing systemic resistance (Lorito et al. 2010). Alternative inoculation sequences (simultaneous inoculation or post-infection *Trichoderma* application) would likely yield different outcomes, as the priming-based mechanism of induced resistance requires establishment of the beneficial microbe prior to pathogen encounter.

## Conclusion

Our data show that root pre-treatments with *Trichoderma* strains (*T. asperellum* ICC012 and *T. gamsii* ICC080) mitigate FW development in melon. In particular, the application of a commercial formulation of these BCAs appear to elicit plant defense responses in the presence of FOM. Particularly, specific hub genes that are upregulated in two key modules (the blue and the green one), which are related to the *Trichoderma-*induced responses of melon roots to the pathogen, may play a pivotal regulatory role in the transcriptional activation of immunity responses. Hence, this study contributes to elucidating the mechanism of action of Trichoderma spp. and provided a roadmap for the further industrial application of *Trichoderma* spp. towards the efficient control of FW in melon.

## Methods and materials

### Plant material and fungal strains

The melon Greek traditional variety 'Xrisi-kefali', which is susceptible to FOM, was chosen as plant material. A virulent FOM strain (isolate 1805) race 0, provided by the Benaki Phytopathological Institute (Athens, Greece), was cultured in potato dextrose agar (PDA) in Petri dishes at 24 °C for 14 days. A commercial fungicide formulation of *Trichoderma* spp. (Remedier WP, Agrology SA, Sindos, Greece), containing selected *Trichoderma* strains (*T. asperellum* ICC012 and *T. gamsii* ICC080), was used to test its biocontrol effect against FOM. The combination of these two strains in a single formulation offers several distinctive advantages compared to other *Trichoderma* strains: i) complementary environmental adaptability, as *T. gamsii* ICC080 demonstrates efficacy at lower temperature compared to *T. asperellum* ICC012; ii) enhanced root colonization capacity; iii) multiple modes of action, including competition for substrate, mycoparasitism, and induction of defense-related genes; and iv) proven efficacy against multiple *Fusarium* species (Lorito et al. 2010; Di Marco et al. 2022; Cesarini et al. 2025).

### *Trichoderma* spp. treatment and pathogen inoculation

Melon seeds were sown in plastic trays filled with sterilized perlite. After their germination (two-cotyledon stage), a batch of the seedlings was transplanted in new sterilized potting soil substrates in plastic pots, which were fully drenched (in a dosage of 15 ml per seedling) with a water solution containing *Trichoderma* spp. formulation (adjusted to a concentration of 2.5 g/L, according to manufacturer recommendation). The other batch of seedlings was transplanted in soil substrates without *Trichoderma* treatment and drenched with sterile distilled water. As above, a second treatment of *Trichoderma* formulation or sterilized dH_2_O in both seedling batches, respectively, was applied again after 2 weeks and 7 days before the FOM inoculation. All seedlings were maintained in greenhouse under standard conditions at 25^°^ C and 80–90% relative humidity with a photoperiod to 16 h day/8 h night. Seedlings of both batches were artificially inoculated with the FOM strain at their four-leaf stage. Roots were gently washed with sterile distilled water, trimmed by pruning them approximately by 1 cm and dipped in the FOM conidial suspension (10^6^ spore/ml) for 30 min. The FOM inoculum was produced as described by Sestili and co-workers (Sestili et al. 2011) and utilized to inoculate: i) roots not previously treated with *Trichoderma* (F treatment), and ii) roots treated with *Trichoderma* (RF treatment). Seedlings untreated with *Trichoderma* and non-inoculated with FOM served as controls (CT treatment). For each of the three treatments (F, RF, CT), three replicates consisting of 10 seedlings each were used. All seedlings were transferred into 10 × 10 cm plastic pots containing sterilized potting soil substrate in a greenhouse at 25^°^ C and 80–90% relative humidity. Seedlings from each treatment were completely randomized. For RNA extraction, root tissues (pooled samples of ten roots per each time point) were collected, snap frozen in liquid nitrogen at three time points (1, 2, 3 days after inoculation) from all the three treatments and stored at −80^°^ C. A total of nine sample treatments were sampled (F1, F2, F3, RF1, RF2, RF3, CT1, CT2, CT3) where numbers denote respectively the day after FOM inoculation.

### Growth promotion effect and disease assessment

To assess the effect of *Trichoderma* spp. pre-treatment against FOM inoculation in roots, ten seedlings per treatment were phenotypically evaluated at eight weeks after treatments (F, RF, CT). Shoot length, stem diameter, and disease incidence were recorded. In addition, a disease index (DI) was used to quantify FW symptoms based on the following empirical scale: 0 = no visible symptoms; 1 = minor leaf yellowing and slight wilting; 2 = moderate leaf yellowing or browning accompanied by partial defoliation; 3 = severe wilting with extensive leaf loss and stem discoloration; and 4 = nearly dead seedlings (McKinney 1923; Sanogo and Zhang 2016). Each seedling was individually scored according to this scale at each evaluation time point, and DI values were calculated as the mean disease severity score per treatment. Disease incidence was calculated as the percentage of fully wilted seedlings. Both disease index and incidence were recorded at four consecutive weekly intervals, starting from the fourth week after pathogen inoculation. All data were subjected to analysis of variance (ANOVA) using the statistical software SPSS statistical software to test for significant differences among treatments. Mean comparisons were performed using Duncan’s multiple range test at a significance level of α = 0.05.

### RNA preparation and transcriptomics

Total RNA was extracted from 27 root samples collected from the three treatments (CT, F, RF) at the three time points by employing three biological replicates. The Monarch Total RNA Miniprep Kit (NEB, Frankfurt, Germany) was used according to the manufacturer’s instructions. Sequencing libraries were constructed using the PT042 NGS RNA Library Prep Set (Novogene Ltd., Cambridge, UK) and RNA-seq was performed using the Illumina Novaseq 6000 platform, where 2 × 150 bp paired-end (PE) reads were generated. Following reads data cleaning using cutadapt (v3.0) (Martin 2011), PE reads were mapped against the melon reference genome assembly (GCF_025177605) and relevant gene models employing the HISAT2 software (v2.0.5) (Kim et al. 2015). The unmapped melon genome reads were subsequently mapped to the FOM strain 26,406 genomic assembly (GCA_000260495) retrieved from ensemblFungi database using the HISAT2 software (v2.0.5) with default settings (Kim et al. 2015). The normalized transcript counts were retrieved using the DESeq2 R package (1.20.0) (Love et al. 2014), while the reads were initially assigned at the transcript level based on the fungal assembly using the htseq-count software (v2.0.2) (Putri et al. 2022).

### Identification of differentially expressed genes in melon

Melon DEGs were retrieved using the DESeq2 R package (1.20.0) (Love et al. 2014) based on an absolute value of log2foldchange ≥ 1 by setting an adjusted *p*-value ≤ 0.05. The software FeatureCounts (v1.5.0-p3) was employed to count the number of reads mapped to each gene (Liao et al. 2014). FPKM values were used to determine gene expression levels. Based on the sample treatments, the DEGs were recorded at nine comparison groups in terms of dual comparisons, namely F1vsCT1, F2vsCT2, F3vsCT3, RF1vsCT1, RF2vsCT2, RF3vsCT3, RF1vsF1, RF2vsF2, RF3vsF3. Venny 2.1 online tool (Oliveros 2007) was used to construct the Venn diagrams to identify the unigenes in a two-step selection process: 1) Venn diagrams were constructed for each treatment comparison among all time points, then 2) among all treatment comparisons resulting from the first step.

###  Screening for key* Trichoderma* spp.* and F. oxysporum f.* sp.* melonis responsive genes in melon by the WGCNA approach*

WGCNA (Weighted Gene Co-Expression Network Analysis) is a systematic biological method used to explore the relationship between genes and associated traits/treatments by identifying co-expressed modules and hub genes within a network. All the identified DEGs (unigenes) were involved in this analysis which was performed using the WGCNA R package (v 1.72–5) (Langfelder and Horvath 2008) in R v4.4.0 (Core Team 2023). Briefly, the pick-Soft Threshold function was executed to determine the appropriate soft-power value (β) which significantly impacts the independence and average connectivity of the co-expression modules. Then the adjacency and topological overlap matrices were created based on the calculation of Pearson’s correlations between each pair of genes. Genes with similar expression profiles were classified into different modules using the dynamic tree cut method based on deepSplit of 2 and minModuleSize of 100 genes, and the modules with more than 80% similarity were merged. A tree diagram was then built by hierarchical clustering, and Spearman rank correlation coefficients were computed between module eigengenes (MEs) and treatments at each time point using the cor function. The modules with a MEs > 0.70 for F or RF treatments was considered as the most decisive modules for our analysis and were further investigated for GO and KEGG pathways enrichment using topGO (v2.2.0) (Alexa and Rahnenführer, 2009) and clusterProfiler (v4.0) (Yu et al. 2012) R packages, respectively. Finally, the intramodular analysis was conducted calculating both Gene Trait Significance (GTS) and module membership (MM), representing the association of individual genes to a given treatment and the correlation between the gene expression profile and module eigengene, respectively. The top 50 hub genes in the critical module were selected for a GTS > 0.75, MM > 0.75, and for the highest |log2FC|, and were chosen as bait genes to construct the module regulatory networks using the Cytoskape CoExpNetViz app (v2.1.5) (Tzfadia et al. 2016). The top 50 hub genes’ expression patterns across the nine comparisons were visualized as a heatmap using pheatmap (v1.0.12) R package. The regulatory networks were then visualized using Cytoscape (v 3.10.2) software (Shannon et al. 2003), where each node represents a gene connected to a different number of genes, the higher the number of connections, the greater the gene importance in response to the correlated treatment.

### Real time quantitative PCR validation of RNA-seq data

Quantitative real-time PCR (RT-qPCR) analysis was performed to assess the relative gene expression of six randomly selected DEGs in order to confirm the RNA-seq data. The LunaScript® RT SuperMix Kit (NEB, Europe) was used to create first-strand cDNA, and the QuantStudio® 5 Real-Time PCR System (Applied Biosystems, Europe) was utilized to conduct quantitative expression analysis using the Luna® Universal qPCR Master Mix (NEB, Europe). Three technical replicates of each PCR reaction were run, and expression levels were normalized to the housekeeping gene LOC103485254 encoding melon actin-7. To determine relative expression levels, the 2^−△△CT^ method was employed (Livak and Schmittgen 2001). The correlation between RNA-seq and RT-qPCR data was confirmed using a linear model. The gene-specific primers used are listed in Table S4.

## Supplementary Information

Additional file 1: Supplementary Figures S1-S9.

Additional file 2: Supplementary Tables 1-10, List of supplementary tables. 

## Data Availability

The datasets generated during the current study are available in the NCBI SRA database below: https://www.ncbi.nlm.nih.gov/, PRJNA1054634.
